# Human epididymis protein 4: a novel predictor of ischemic cardiomyopathy

**DOI:** 10.1186/s12872-021-02319-5

**Published:** 2021-10-21

**Authors:** Yi Tang, Yinzhen Wang, Xiaoping Xu, Laura Yan Tu, Pei Huang, Xiaoyan Yang, Lihua Li, Juan Wu, Yan Zhang, Qinghua Fu, Yingli Yu, Zhaofen Zheng, Lixia Song, Yi Zhang

**Affiliations:** 1 Department of Cardiology, Hunan Provincial People’s Hospital, The First Affiliated Hospital of Hunan Normal University, Clinical Medicine Research Center of Heart Failure of Hunan Province, Hunan Normal University, Changsha, 410005 China; 2 Department of Gastroenterology, Hunan Provincial People’s Hospital, The First Affiliated Hospital of Hunan Normal University, Hunan Normal University, Changsha, 410005 China; 3 Department of Medicine, Austin Health, Melbourne, VIC 3084 Australia; 4 Institute of Traditional Chinese Medicine, Tianjin University of Traditional Chinese Medicine, Tianjin, 300193 China

**Keywords:** Human epididymis protein 4, Ischemic cardiomyopathy, Predictor

## Abstract

**Background:**

The prognostic value of human epididymis protein 4 (HE4) in patients with ischemic cardiomyopathy (ICM) is unknown.

**Methods:**

A total of 103 patients with ICM were prospectively enrolled in this study from Hunan Provincial People’s Hospital between February 2019 and June 2019. All patients were tested for HE4 levels at baseline and follow-up. Endpoints of the study included cardiovascular death and heart failure-related hospitalization.

**Results:**

A total of 96 patients with ICM were included for analysis. After a mean follow-up period of 263 (153–313) days, cardiovascular events were observed in 45 patients. Serum HE4 levels in patients with events were significantly higher than those in patients without events [188.70 (113.35–326.82) pmol/L versus 92.90 (61.50–123.20) pmol/L, *P* < 0.001]. Multivariate Cox regression analysis revealed that HE4 [χ^2^: 9.602, hazard ratio (HR): 1.003, 95% confidence interval (CI): 1.001–1.005, *P* = 0.002] and age [χ^2^: 4.55, HR: 1.044, 95% CI: 1.003–1.085, *P* = 0.033] were independent predictors of events. After adjusting for age and sex, the risk of events in patients with HE4 > 100.2 pmol/L was higher than that in patients with HE4 ≤ 100.2 pmol/L [HR: 3.372, 95% CI: 1.409–8.065, *P* < 0.001].

**Conclusion:**

HE4 is an independent predictor of cardiovascular death and heart failure-related rehospitalization in patients with ICM.

## Introduction

Heart failure comprises a group of clinical syndromes that reflect advanced stages of various heart diseases. A total of 1–2% of the adult population in developed countries has heart failure [1]. Following the diagnosis of heart failure, survival estimates are only 50% at 5 years [2]. Heart failure is a global public health problem, and early diagnosis, risk stratification and treatment are especially important. In addition to N-terminal prohormone of brain-type natriuretic peptide (NT-proBNP), several new biomarkers, such as fibroblast growth factor 23 (FGF23), fibrotic biomarkers such as galectin-3 (Gal-3) and soluble suppression of tumorigenicity 2 (sST2), are helpful in the diagnosis, risk stratification and prognostication of heart failure [3–5].

Human epididymis protein 4 (HE4), also known as whey acidic protein four-disulfide core domain 2 (WFDC2), is expressed in various tissues, such as the kidney and ovary. It plays an important role during innate immune defense and tumorigenesis [6]. HE4 is a protease inhibitor that also suppresses the activities of multiple proteases, such as serine proteases and matrix metalloproteinases. HE4 was proven to be involved in renal fibrosis [7]. HE4 has also been demonstrated to be a new biomarker of acute and chronic left heart failure [8, 9]. Our study also demonstrated that HE4 is an independent predictor of clinical deterioration in idiopathic pulmonary arterial hypertension (IPAH) patients with right heart failure [10]. As HE4 has been proven to be associated with processes of renal fibrosis, it was speculated that HE4 may play a role in myocardial fibrosis.

Ischemic cardiomyopathy (ICM) is the primary type of heart failure, accounting for 36–52% of clinical syndromes with specific pathological and physiological mechanisms. Acute and chronic ischemia can result in myocardial stunning or myocardial necrosis, with subsequent myocardial hypertrophy and fibrosis, leading to heart failure [11, 12]. Whether HE4 predicts the prognosis of patients with ICM is unknown. This study aims to explore the prognostic value of HE4 in patients with ICM.

## Materials and methods

### Study population

From February 2019 to June 2019, 103 adult patients diagnosed with ICM admitted to Hunan Provincial People's Hospital were prospectively enrolled in this study. ICM was defined according to the following criteria: (1) clear evidence of myocardial ischemia or myocardial infarction. At least one of the following conditions need to be fulfilled: 1. coronary arteriography demonstrates more than 75% stenosis in the left main coronary artery or left anterior descending artery or two vessels or more with greater than 75% stenosis, meanwhile, resting electrocardiogram/dynamic electrocardiogram /electrocardiogram treadmill exercise test or echocardiography/dobutamine stress echocardiography indicates myocardial ischemia, 2. patients underwent coronary revascularization or had a history of myocardial infarction[12]; (2) enlargement of the left ventricle due to myocardial ischemia or myocardial infarction (echocardiography revealed that left ventricular end-diastole diameter ≥ 50 mm for women or ≥ 55 mm for men); and (3) clinical manifestations of heart failure. Patients with the following conditions were excluded: (1) ventricular septal perforation, ventricular aneurysm or papillary muscle dysfunction leading to mitral regurgitation; (2) women who were pregnant; and (3) patients with a history of cancer. This research was conducted in compliance with the Declaration of Helsinki and was approved by the Ethics Committee of Hunan Provincial People's Hospital (2018-50). Informed consent was obtained from all enrolled patients.

### Clinical assessment and echocardiography

Basic demographic data, such as age, sex, biochemical tests and New York Heart Association Functional Classification (NYHA-FC), were collected at baseline. The Modification of Diet in Renal Disease (MDRD) formula was used to calculate the estimated glomerular filtration rate (eGFR). eGFR(ml/min/1.73m^2^) = 30,849 × [Scr(μmol/L)]^−1.154^ × (age)^−0.203^ × (0.742 if patient is female). NT-proBNP was determined at baseline using the chemiluminescence immunoassay method (Wantaikairui, XiaMen, China) in the Department of Laboratory Medicine of Hunan Provincial People's Hospital.

All patients underwent echocardiographic examination within 3 days after admission using GE Vivid E9 machines with a 3.5 MHz probe (General Electric Company, U.S.). Echocardiographic examination was performed according to the American Society of Echocardiography recommendations [13]. A parasternal long-axis M-mode image was acquired to measure the left ventricular end diastolic diameter (LVEDD). The left ventricular ejection fraction (LVEF) was measured by a modified version of Simpson's biplane method. left ventricular diastolic function was determined using a combination of mitral spectral pulse-wave Doppler and tissue Doppler of the mitral annulus according to the American Society of Echocardiography recommendations [14]. A dobutamine stress echocardiogram was performed to evaluate whether myocardial ischemia existed in some patients.

### Follow-up

Patients were followed up in the first, third, sixth, and twelfth months and every 6 months following discharge from the hospital. The end point events were defined as the time from the date of blood sampling to cardiovascular death and/or heart failure-related rehospitalization. The follow-up time was calculated from the time of blood sampling to February 15, 2020.

### HE4 measurement

Peripheral venous blood samples were collected the following morning after admission. Blood samples were centrifuged at 1000 rpm for 15 min, and the upper serum was frozen and stored at -80℃. Serum levels of HE4 were measured using a chemiluminescence microparticle immunoassay (Abbott, Germany) in the Department of Nuclear Medicine of Hunan Provincial People's Hospital. The detectable dose of human HE4 ranged from 15 to 1500 pmol/L. The investigator was blinded with respect to clinical data.

### Statistical analysis

The data are presented as the mean ± standard deviation ($\overline{x} \pm s$) for continuous variables with normal distribution, as interquartile ranges (IQRs) for continuous variables with nonnormal distribution, and as percentages for categorical variables. Statistical differences between two groups were analyzed using an independent Student’s t-test for normally distributed variables and the Mann–Whitney U test for nonnormally distributed variables. Chi-square test was used for categorical variables. Pearson or Spearman correlation coefficients were used as appropriate to explore correlations between HE4 and other variables. The optimal cutoff value of HE4 was determined by receiver operating characteristic (ROC) curve analysis. Univariate Cox regression analysis was used to explore the predictive value of each variable for events, and then a forward stepwise multivariate Cox regression model was further utilized to explore independent predictors. Kaplan–Meier plots with log-rank tests were used to illustrate outcomes of patients grouped by the optimal cutoff value of HE4. ROC analysis was performed using Med Calc 13.0 (Med Calc Software), and other statistical analyses were performed using SPSS version 19.0 (SPSS, Inc.). A 2-sided *P* < 0.05 was considered statistically significant.

## Results

### Baseline characteristics

A total of 103 patients with ICM were enrolled, 7 of whom were lost to follow-up and were excluded from the analysis. Among the remaining 96 patients, 63 (65.6%) were males. The median age was 71 (65–77) years. The median left ventricular ejection fraction (LVEF) was 33 (29–42)%. Twenty-eight patients (29.2%) presented with NYHA-FC II, 40 patients (41.7%) presented with NYHA-FC III symptoms, and 28 patients (29.2%) presented with NYHA-FC IV symptoms. The median follow-up was 263 (153–313) days. Forty-five patients (47%) experienced events, 12 patients died, and 33 patients were hospitalized due to heart failure (Table 1). Three patients received cardiac resynchronization therapy, and 2 patients received implantable cardioverter defibrillators.

**Table 1:** Baseline characteristics of patients with ischemic cardiomyopathy with and without events

Variables	Events group (n = 45)	No events group (n = 51)	Total (n = 96)	*P-*value
Age (y)	75 (70–81)	68 (61–73)	71 (65–77)	< 0.001
Male, n (%)	28 (62.2%)	35 (68.6%)	63 (65.6%)	0.510
BMI (kg/m^2^)	23.35 ± 3.48	24.09 ± 3.07	23.78 ± 3.24	0.326
Hypertension, n (%)	28 (62.2%)	30 (58.8%)	58 (60.4%)	0.734
Atrial fibrillation, n (%)	8 (17.8%)	4 (7.8%)	12 (12.5%)	0.142
Diabetes, n (%)	15 (33.3%)	19 (37.3%)	34 (35.4%)	0.688
NYHA-FC				0.006
II, n (%)	7 (15.6%)	21 (41.2%)	28 (29.2%)	
III/IV, n (%)	38 (84.4%)	30 (58.8%)	68 (70.9%)	
LVEF (%)	32 (27–42)	33 (30–42)	33 (29–42)	0.368
LVEDD/BSA (mm/m^2^)	35.7(33.8–39.9)	35.6(33.6–38.5)	35.6(33.6–39.2)	0.51
NT- proBNP (ng/L)	7084.0 (3147.0–15,175.5)	1874.5 (937.7–4902.7)	3682.0 (1247.0–21,403.4)	< 0.001
HE4 (pmol/L)	188.7 (113.3–326.8)	92.9 (61.50–123.2)	117.6 (80.1–196.8)	< 0.001
eGFR (mL/min/1.73 m^2^)	53.72 ± 23.48	77.04 ± 25.48	66.11 ± 27.09	< 0.001
WBC (10^9^/L)	6.74 (5.35–8.85)	6.26 (5.33–7.15)	6.48 (5.34–7.84)	0.187
LDL-C(mmol/L)	2.44 (1.88–2.79)	2.33 (1.73–3.36)	2.37 (1.85–3.07)	0.519
ALT (U/L)	16.5 (11.0–42.8)	18.5 (13.8–31.7)	17.6 (12.2–35.4)	0.436
HbA_1c_ (%)	5.75 (5.50–6.50)	6.00 (5.53–6.93)	5.90 (5.50–6.70)	0.389
CK-MB(U/L)	13.0 (0.5–22.0)	14.5 (10.0–20.0)	13.0 (0–20.0)	0.887
PCI, n (%)	20 (44.4%)	30 (58.8%)	50 (52.1%)	0.159
Diuretics, n (%)	32 (71.1%)	30 (58.8%)	62 (64.6%)	0.209
ACEI/ARB/ARNI, n (%)	34 (81.0%)	46 (90.2%)	80 (86.0%)	0.201
β-Blocker, n (%)	37 (82.2%)	47 (92.2%)	84 (87.5%)	0.142

*ACEI* angiotensin-converting enzyme inhibitor, *ALT* alanine aminotransferase, *ARB* angiotensin receptor blocker, *ARNI* angiotensin receptor and enkephalinase inhibitor, *BMI* body mass index, *CK-MB* creatine kinase isoenzyme-MB, *eGFR* estimated glomerular filtration rate, *HbA1c* hemoglobin A1c, *HE4* human epididymis protein, *LDL* low-density lipoprotein, *LVEDD/BSA* left ventricular end diastolic dimension/body surface area, *LVEF* left ventricular ejection fraction, *MDRD* Modification of Diet in Renal Disease, *NT-proBNP* N-terminal prohormone of B-type natriuretic peptide, *NYHA-FC* New York Heart Association Functional Classification, *PCI* percutaneous coronary intervention, *WBC* white blood cells

**Fig. 1:**
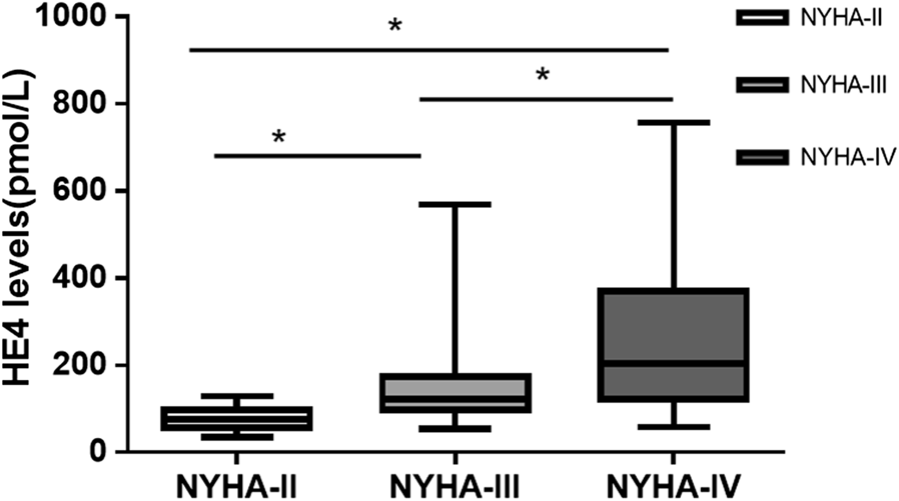
HE4 levels in patients with ischemic cardiomyopathy (ICM) with NYHA-FC II-IV. *P < 0.05

As NYHA-FC increased, HE4 levels became significantly higher (NYHA-FC II: 75.30 pmol/L, IQR 57.18–97.75; NYHA-FC III: 121.90 pmol/L, IQR 98.25–174.85; NYHA-FC IV: 203.60 pmol/L, IQR 121.93–317.18) (Fig. 1). HE4 levels correlated positively with NT-proBNP (r = 0.602, *P* < 0.001) and negatively with eGFR (r = − 0.697, *P* < 0.001). No significant correlation was observed between HE4 and left ventricular ejection fraction (r = − 0.082, *P* = 0.426).

### Comparison between groups

Patients with events had a similar sex distribution and body mass index (BMI) compared to patients without events. There was no difference in complications or treatment regimen using angiotensin-converting enzyme inhibitor (ACEI), angiotensin receptor blocker (ARB), angiotensin receptor-neprilysin inhibitor (ARNI), β-blockers or percutaneous coronary intervention (PCI) between patients with and without events. However, patients with events exhibited less favorable NYHA-FC and renal function, older age, and higher NT-proBNP and HE4.

### HE4 and clinical outcomes

In univariate analysis, HE4, NT-proBNP, eGFR, NYHA-FC and age were all significant predictors of events (Table 2). Multivariable forward stepwise Cox analysis was performed including HE4, NT-proBNP, eGFR, NYHA-FC and age. The results showed that HE4 [χ^2^: 9.602, hazard ratio (HR): 1.003, 95% confidence interval (CI): 1.001–1.005, *P* = 0.002] and age [χ^2^, 4.557; HR: 1.044, 95% CI: 1.003–1.085, *P* = 0.033] were independent predictors of events (Table 3). The forward stepwise and backward stepwise analyses yielded the same results.

**Table 2:** Univariate Cox analysis of proportional risks for events in patients with ischemic cardiomyopathy

Variables	χ^2^	HR (95% CI)	*P*-value
HE4	26.605	1.004 (1.003–1.006)	< 0.001
Log_10_NT-proBNP	18.228	3.615 (2.004–6.520)	< 0.001
eGFR (MDRD)	13.784	0.978 (0.967–0.996)	< 0.001
NYHA-FC	9.648	1.903 (1.268–2.855)	0.002
Age	14.579	1.071 (1.034–1.110)	< 0.001
LVEF	1.258	0.984 (0.956–1.012)	0.262

*CI* confidence interval, *eGFR* estimated glomerular filtration rate, *HE4* human epididymis protein 4, *HR* hazard ratio, *LVEF* left ventricular ejection fraction, *MDRD* Modification of Diet in Renal Disease, *NT-proBNP* N-terminal prohormone of B-type natriuretic peptide, *NYHA-FC* New York Heart Association Functional Classification

**Table 3:** Multivariable forward stepwise Cox analysis of proportional risks for events in patients with ischemic cardiomyopathy

Variables	β	SE	χ^2^	HR (95%CI)	*P*-value
HE4	0.003	0.001	9.602	1.003(1.001–1.005)	0.002
Age	0.043	0.02	4.557	1.044(1.003–1.085)	0.033

*HE4* human epididymis protein 4

**Fig. 2:**
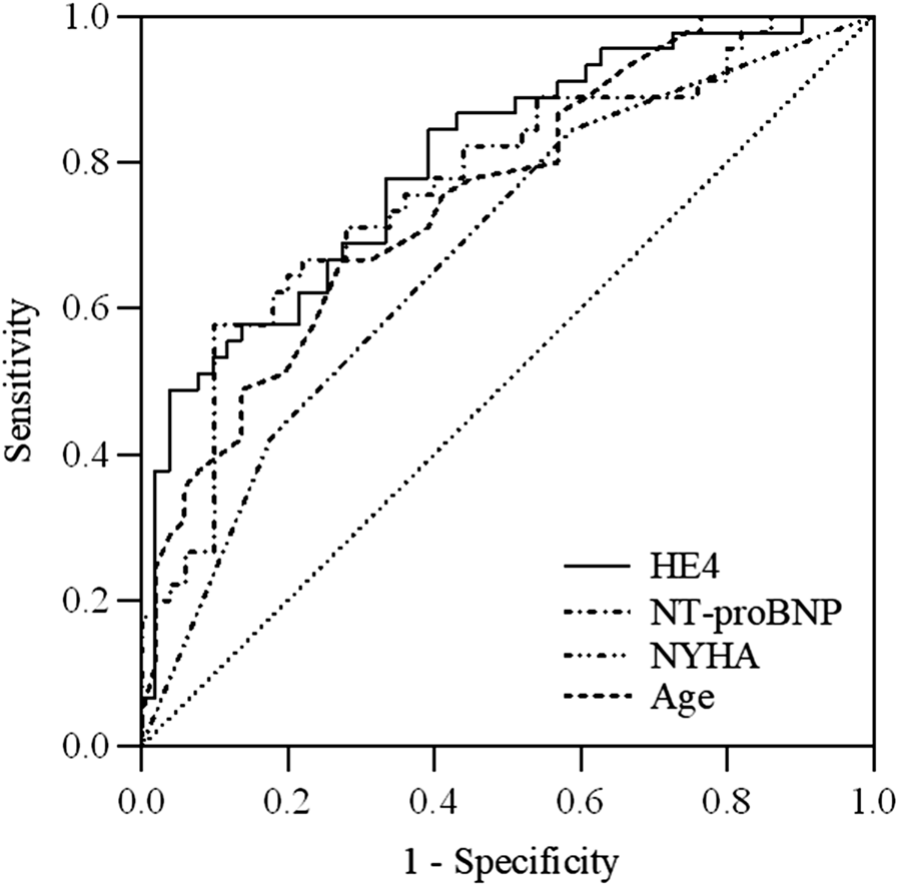
Receiver operating characteristic curve showing the sensitivity and specificity of HE4, NT-proBNP, age, and NYHA to predict cardiovascular events. HE4 human epididymis protein 4, NT-proBNP: N-terminal prohormone of B-type natriuretic peptide, NYHA-FC: New York Heart Association Functional Classification

The ROC analysis showed that HE4 [area under the curve (AUC) = 0.800] was the best predictor for events, followed by NT-proBNP (AUC = 0.766), age (AUC = 0.753), and NYHA-FC (AUC = 0.678) (Table 4 and Fig. 2).

**Table 4:** Receiver operating characteristic curve analysis of variables in predicting events

Variables	AUC	95% CI	Sensitivity (%)	Specificity (%)
HE4	0.800	0.712–0.888	84.44	60.78
NT-proBNP	0.766	0.669–0.862	57.78	90.00
Age	0.753	0.658–0.849	66.67	72.55
NYHA-FC	0.678	0.570–0.785	84.44	41.18

*AUC* area under the curve, *CI* confidence interval, *HE4* human epididymis protein, *eGFR* estimated glomerular filtration rate, *NYHA-FC* New York Heart Association Functional Classification

**Fig. 3:**
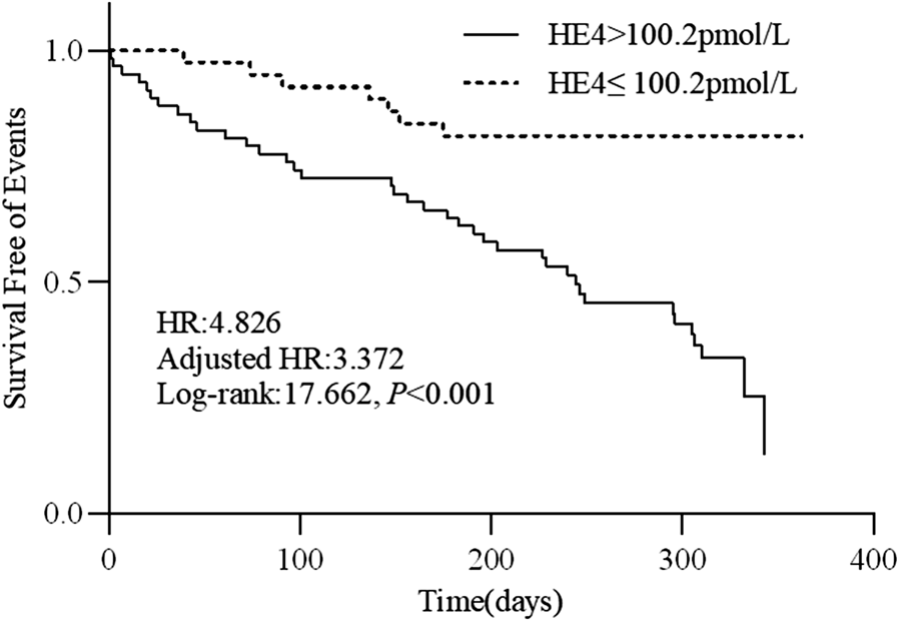
Kaplan–Meier analysis of human epididymis protein (HE4) for cardiovascular death and heart failure rehospitalization. Adjusted HR indicates hazard ratio (HR) adjusted for age and gender

Patients with HE4 > 100.2 pmol/L exhibited a worse survival rate than those with HE4 ≤ 100.2 pmol/L (log-rank rest: 17.662,* p* < 0.001). The unadjusted HR was 4.826 (95% CI: 2.148–10.846), and the HR after adjustment for age and sex was 3.372 (95% CI: 1.409–8.065). The Kaplan–Meier curves are shown in Fig. 3.

## Discussion

To our knowledge, this is the first study to examine HE4 as an independent predictor of cardiovascular death and heart failure rehospitalization in patients with ICM. Herein, we revealed that serum HE4 levels in patients with events were significantly higher than those in patients without events. HE4 was positively correlated with NT-proBNP, and its levels were significantly increased as NYHA-FC increased. HE4 may be a novel biomarker of heart failure in patients with ICM, assisting in the diagnosis of heart failure. The significant increase in HE4 may indicate a poor prognosis for patients with ICM, and patients with high HE4 levels may benefit from early intensive therapy.

HE4 is a protease inhibitor that is involved in renal fibrosis by suppressing the activity of multiple proteases, such as serine proteases and matrix metalloproteinases, and reducing the degradation of collagen I [7]. Studies have shown that HE4 is a fibroblast-derived mediator of renal fibrosis in a mouse model and is elevated in patients with chronic kidney disease [7, 15]. Several studies report a strong correlation between HE4 and creatinine [15–17]. Our study showed deteriorating renal function with increasing HE4 levels. This might be ascribed to HE4 being involved in renal fibrosis.

It has also been reported that serum HE4 levels are elevated in patients with left heart failure and right heart failure and exhibit a significant correlation with galectin-3, a biomarker of myocardial fibrosis [9, 10]. Our study revealed that HE4 levels increased as NYHA-FC deteriorated, and it had a positive correlation with NT-proBNP. More importantly, we found that HE4 was an independent predictor of cardiovascular death and heart failure rehospitalization in patients with ICM, which was in accordance with the results of previous studies [8–10]. ICM consists of a spectrum of pathophysiological states that relate to perfusion contraction matching and mismatching. Myocardial fibrosis is the primary characteristic of ICM [12]. As HE4 is involved in renal fibrosis by suppressing the activity of multiple proteases and reducing the degradation of collagen I, we speculate that HE4 may also play a key role in myocardial fibrosis via the same biological mechanism in patients with ICM.

Our study has several limitations. First, HE4 levels were only measured at baseline, and changes in HE4 levels after short- or long-term treatment are unknown. Second, the sample size of our study was relatively small, and a multicenter, large-scale sample will be required to further validate these results.

## Conclusion

HE4 levels are associated with the severity of heart failure and independently predict cardiovascular death and heart failure rehospitalization in patients with ischemic cardiomyopathy.

## Data Availability

The datasets used and analyzed during the present study are available from the corresponding author on reasonable request.

## References

[1] Mosterd A, Cost B, Hoes AW, de Bruijne MC, Deckers JW, Hofman A, Grobbee DE (2001). The prognosis of heart failure in the general population: the Rotterdam Study. Eur Heart J.

[2] Cowie MR, Wood DA, Coats AJ, Thompson SG, Suresh V, Poole-Wilson PA, Sutton GC (2000). Survival of patients with a new diagnosis of heart failure: a population based study. Heart (British Cardiac Society).

[3] Poelzl G, Trenkler C, Kliebhan J, Wuertinger P, Seger C, Kaser S, Mayer G, Pirklbauer M, Ulmer H, Griesmacher A (2014). FGF23 is associated with disease severity and prognosis in chronic heart failure. Eur J Clin Invest.

[4] de Boer RA, van der Velde AR, Mueller C, van Veldhuisen DJ, Anker SD, Peacock WF, Adams KF, Maisel A (2014). Galectin-3: a modifiable risk factor in heart failure. Cardiovasc Drugs Ther.

[5] Bayes-Genis A, Zhang Y, Ky B (2015). ST2 and patient prognosis in chronic heart failure. Am J Cardiol.

[6] Galgano MT, Hampton GM, Frierson HF (2006). Comprehensive analysis of HE4 expression in normal and malignant human tissues. Mod Pathol.

[7] LeBleu VS, Teng Y, O'Connell JT, Charytan D, Müller GA, Müller CA, Sugimoto H, Kalluri R (2013). Identification of human epididymis protein-4 as a fibroblast-derived mediator of fibrosis. Nat Med.

[8] de Boer RA, Cao Q, Postmus D, Damman K, Voors AA, Jaarsma T, van Veldhuisen DJ, Arnold WD, Hillege HL, Silljé HH (2013). The WAP four-disulfide core domain protein HE4: a novel biomarker for heart failure. JACC Heart Fail.

[9] Piek A, Meijers WC, Schroten NF, Gansevoort RT, de Boer RA, Silljé HH (2017). HE4 serum levels are associated with heart failure severity in patients with chronic heart failure. J Cardiac Fail.

[10] Jin Q, Tang Y, Liu Z, Xie W, Luo Q, Zhao Z, Zhao Q, Huang Z, Yu X, Yan L (2020). Serum human epididymis protein 4 level as a predictor of clinical worsening in idiopathic pulmonary arterial hypertension: a pilot study. BMC Cardiovasc Disord.

[11] Fox KF, Cowie MR, Wood DA, Coats AJ, Gibbs JS, Underwood SR, Turner RM, Poole-Wilson PA, Davies SW, Sutton GC (2001). Coronary artery disease as the cause of incident heart failure in the population. Eur Heart J.

[12] Briceno N, Schuster A, Lumley M, Perera D (2016). Ischaemic cardiomyopathy: pathophysiology, assessment and the role of revascularisation. Heart (British Cardiac Society).

[13] Lang RM, Badano LP, Mor-Avi V, Afilalo J, Armstrong A, Ernande L, Flachskampf FA, Foster E, Goldstein SA, Kuznetsova T (2015). Recommendations for cardiac chamber quantification by echocardiography in adults: an update from the American Society of Echocardiography and the European Association of Cardiovascular Imaging. J Am Soc Echocardiogr.

[14] Nagueh SF, Smiseth OA, Appleton CP, Byrd BF, Dokainish H, Edvardsen T, Flachskampf FA, Gillebert TC, Klein AL, Lancellotti P (2016). Recommendations for the evaluation of left ventricular diastolic function by echocardiography: an update from the American Society of Echocardiography and the European Association of Cardiovascular Imaging. J Am Soc Echocardiogr.

[15] Wan J, Wang Y, Cai G, Liang J, Yue C, Wang F, Song J, Wang J, Liu M, Luo J (2016). Elevated serum concentrations of HE4 as a novel biomarker of disease severity and renal fibrosis in kidney disease. Oncotarget.

[16] Yuan T, Li Y (2017). Human epididymis protein 4 as a potential biomarker of chronic kidney disease in female patients with normal ovarian function. Lab Med.

[17] Wang L, Sun Y, Cai X, Fu G (2018). The diagnostic value of human epididymis protein 4 as a novel biomarker in patients with renal dysfunction. Int Urol Nephrol.

